# Supplementary material for: Inhibition of the Niemann-Pick C1 protein is a conserved feature of multiple strains of pathogenic mycobacteria
Source: Nat Commun. 2022 Sep 9;13:5320. doi: 10.1038/s41467-022-32553-0 (PMC9463166; doi:10.1038/s41467-022-32553-0)
Supplement: Supplementary file 1 — Supplementary Information [file 41467_2022_32553_MOESM1_ESM.pdf]

Supplementary Information

Inhibition of the Niemann-Pick C1 protein is a conserved feature of multiple strains of pathogenic mycobacteria.

Weng et al.

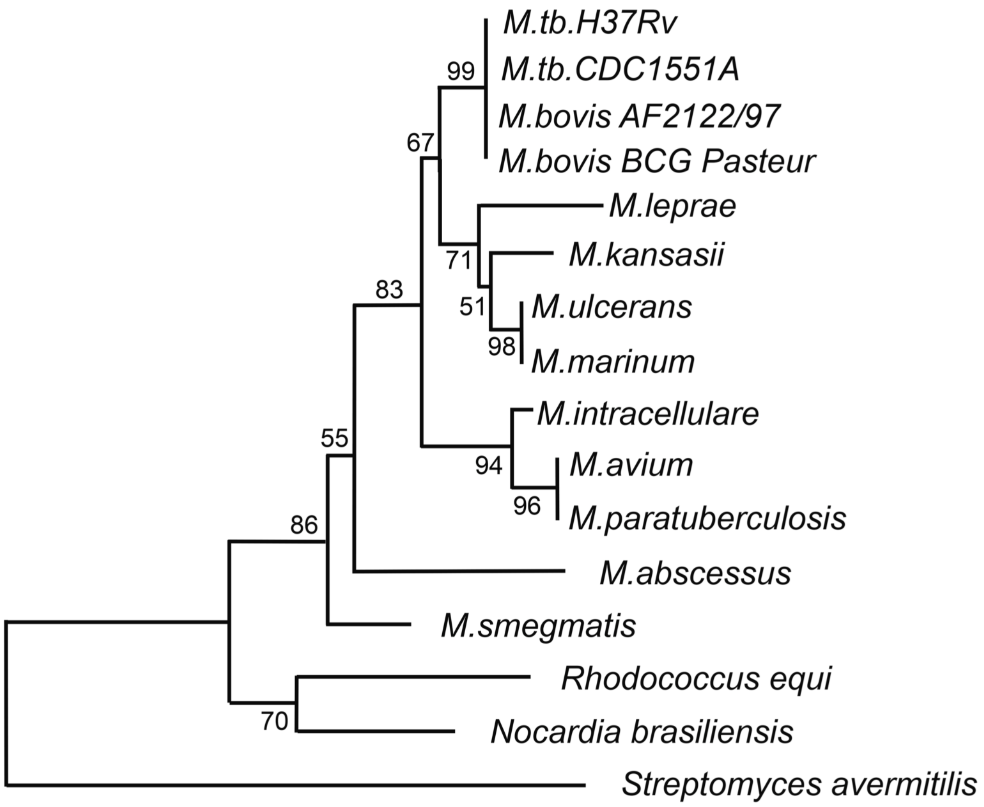

Supplementary Figure 1: Schematic of phylogenetic relationship of specific mycobacterial species. Phylogenetic tree of mycobacteria based upon the relatedness of the iron-regulated protein HupB[1].

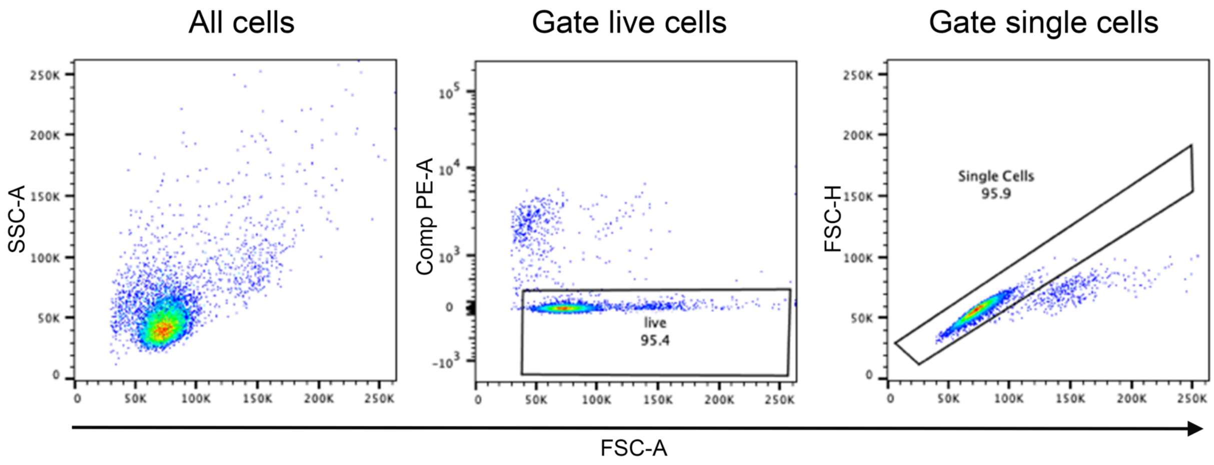

Supplementary Figure 2: FACS gating strategy for LysoTracker™ staining of RAW 264.7 MΦ. Representative dot plots of RAW 264.7 cells gated for live cells (PI negative, centre panel)) and live singlets (right panel).

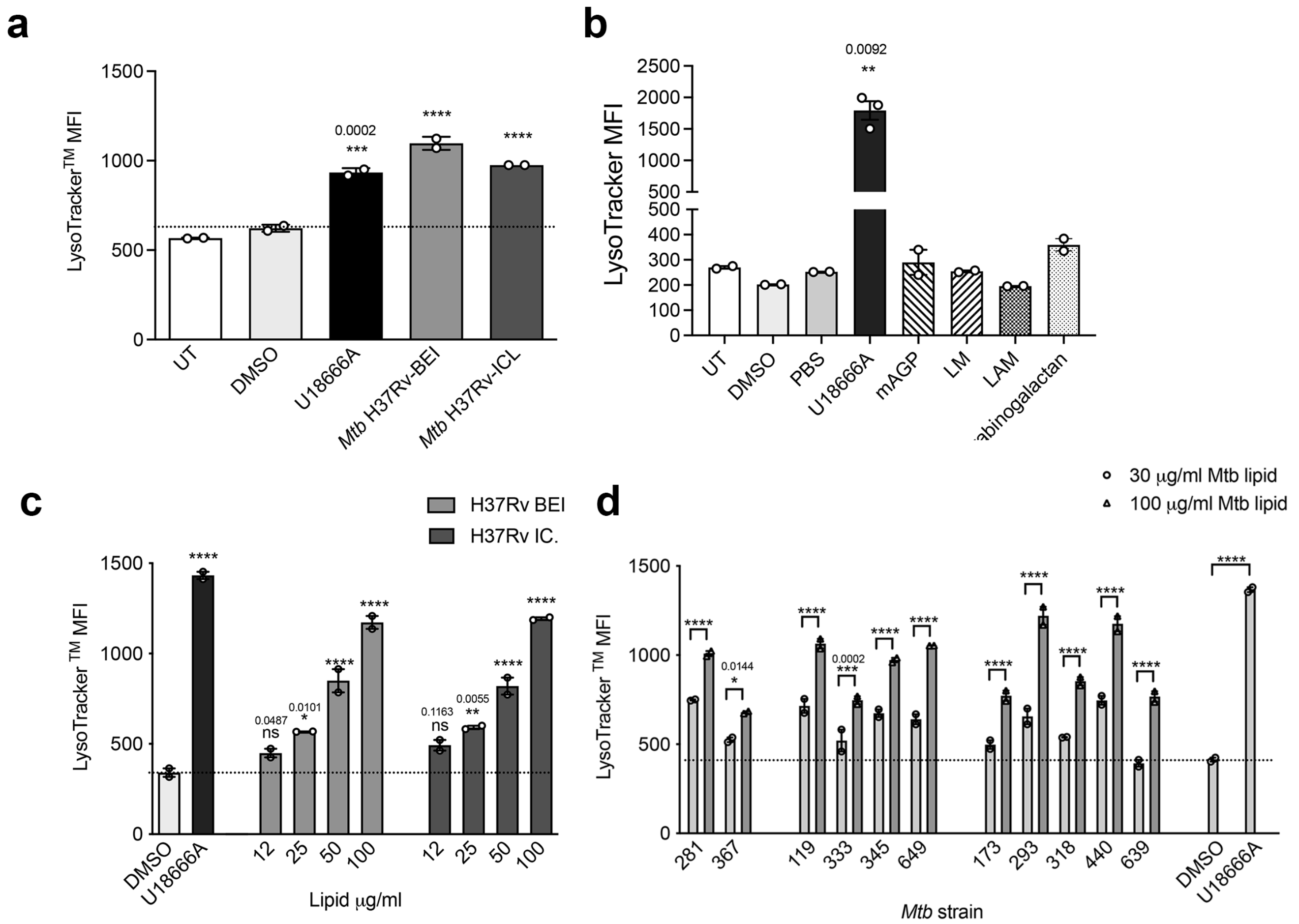

~

Supplementary Figure 3: Dose-dependent effects of *Mtb* H37Rv lipid extracts from two independent sources, *Mtb* clinical strains and specific *Mtb* components on LysoTracker™ intensities of RAW 264.7 MΦ. Panel a. LysoTracker™ intensity values of RAW 264.7 MΦ treated with 100µg/ml H37Rv lipid extract sourced from BEI or Imperial College for 48h. Data are mean ± SEM, N= minimum of 2 replicates per sample. Statistical analysis, 1-way ANOVA, **** p<0.0001, all other significance values as indicated. Panel b. LysoTracker™ intensity values of RAW 264.7 MΦ untreated, vehicle treated, U18666A treated and incubated with purified mycolylarabinogalactan-peptidoglycan (mAGP), lipomannan (LM) lipoarabinomannan (LAM) or arabinogalactan at 50 µg/ml for 48h (all sourced from BEI). Data are mean ± SEM, N= minimum of 2 replicates per sample. Statistical analysis, 1-way ANOVA, statistical significance as indicated, otherwise not significant. Panel c. LysoTracker™ intensity values of RAW 264.7 MΦ treated with different amounts (12, 25, 50 and 100µg/ml) of H37Rv lipid extract sourced from BEI or Imperial College for 48h. Data are mean ± SEM, N= minimum of 2 replicates per sample. Statistical analysis, 1-way ANOVA, **** p<0.0001, other significance values as indicated. Panel d. Dose-dependent increase in LysoTracker™ intensities of RAW 264.7 MΦ in response to incubation with vehicle, U18666A or lipid extracts of *Mtb* clinical strains at 30 µg/ml and 100 µg/ml. Data are mean ± SEM, N= minimum of 2 replicates per sample. Statistical analysis, 1-way ANOVA. **** p<0.0001, all other significance values as indicated. All data are representative of a minimum of two independent experiments. Source data are provided as a Source data file.

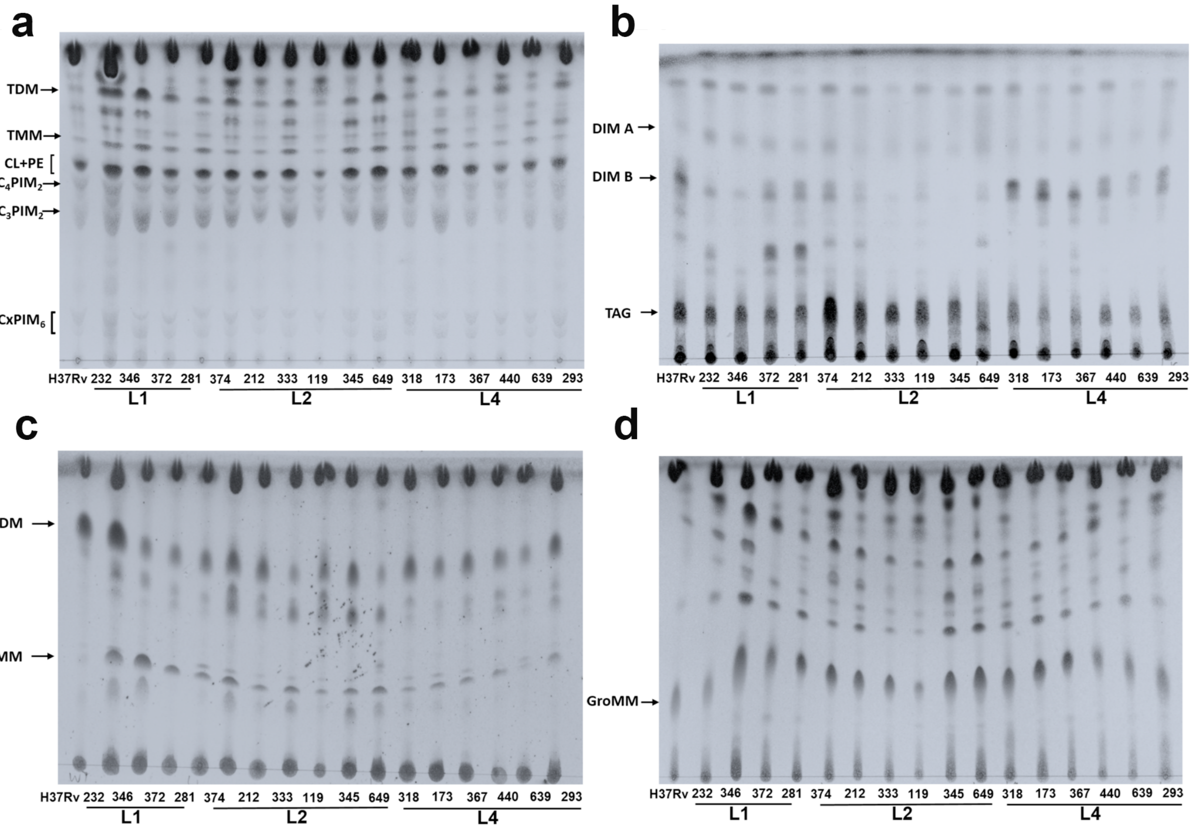

a
TDM
TMM
CL+PE
AC4PIM2
AC3PIM2
ACxPIM6
H37Rv 232 346 372 281 374 212 333 119 345 649 318 173 367 440 639 293
L1
L2
L4
b
DIM A
DIM B
TAG
H37Rv 232 346 372 281 374 212 333 119 345 649 318 173 367 440 639 293
L1
L2
L4
c
TDM
TMM
H37Rv 232 346 372 281 374 212 333 119 345 649 318 173 367 440 639 293
L1
L2
L4
d
GroMM
H37Rv 232 346 372 281 374 212 333 119 345 649 318 173 367 440 639 293
L1
L2
L4

e

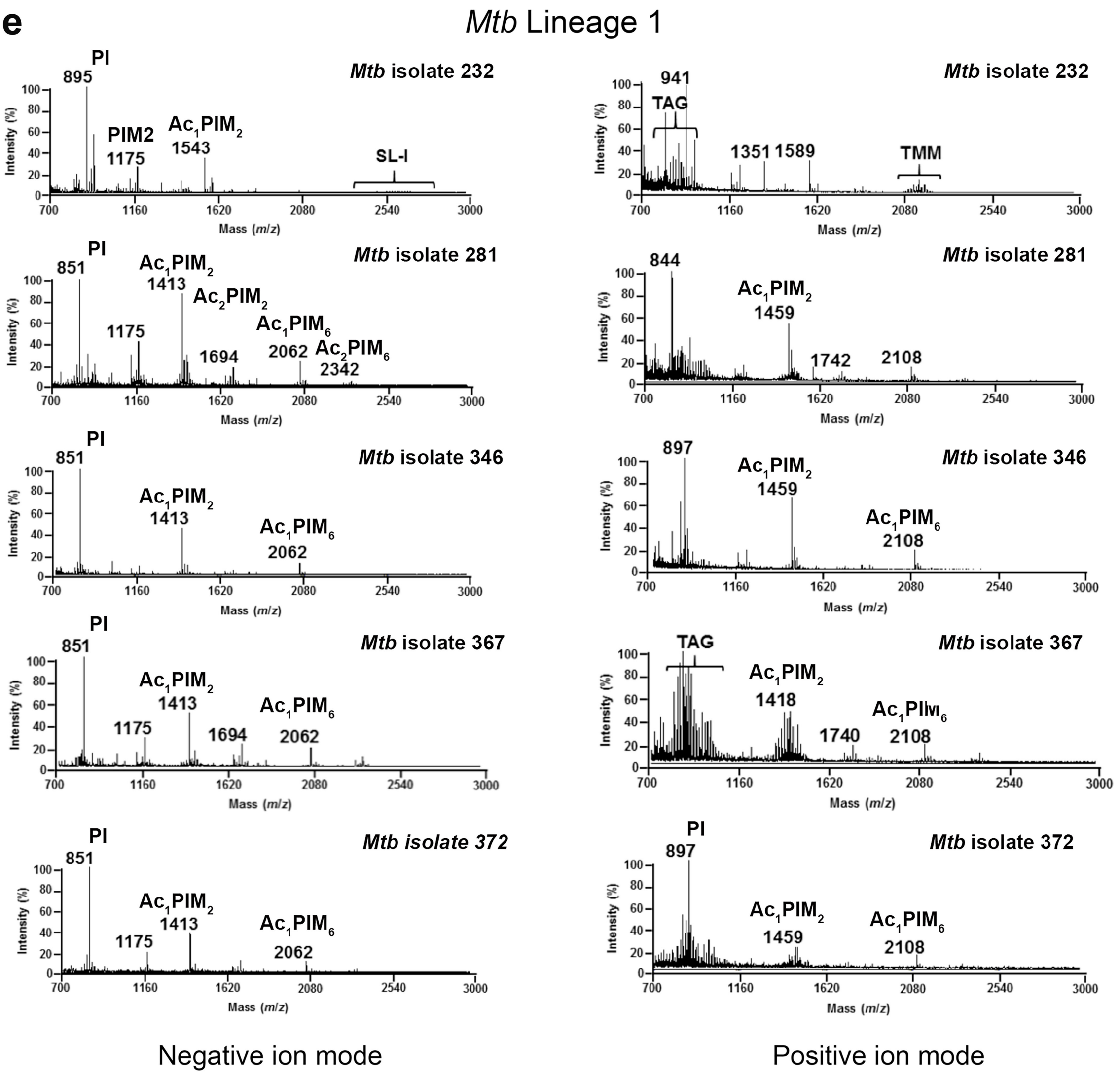

Mtb Lineage 1
Mtb isolate 232
PI
895
PIM2
1175
Ac1PIM2
1543
SL-I
941
TAG
1351
1589
TMM
Mtb isolate 281
851
1175
Ac1PIM2
1413
Ac2PIM2
1694
Ac1PIM6
2062
Ac2PIM6
2342
844
1459
1742
2108
Mtb isolate 346
1413
2062
897
1459
2108
Mtb isolate 367
1175
1413
1694
2062
1418
1740
2108
Mtb isolate 372
851
1175
1413
2062
897
1459
2108
Intensity (%)
Mass (m/z)
Negative ion mode
Positive ion mode

f *Mtb* Lineage 2

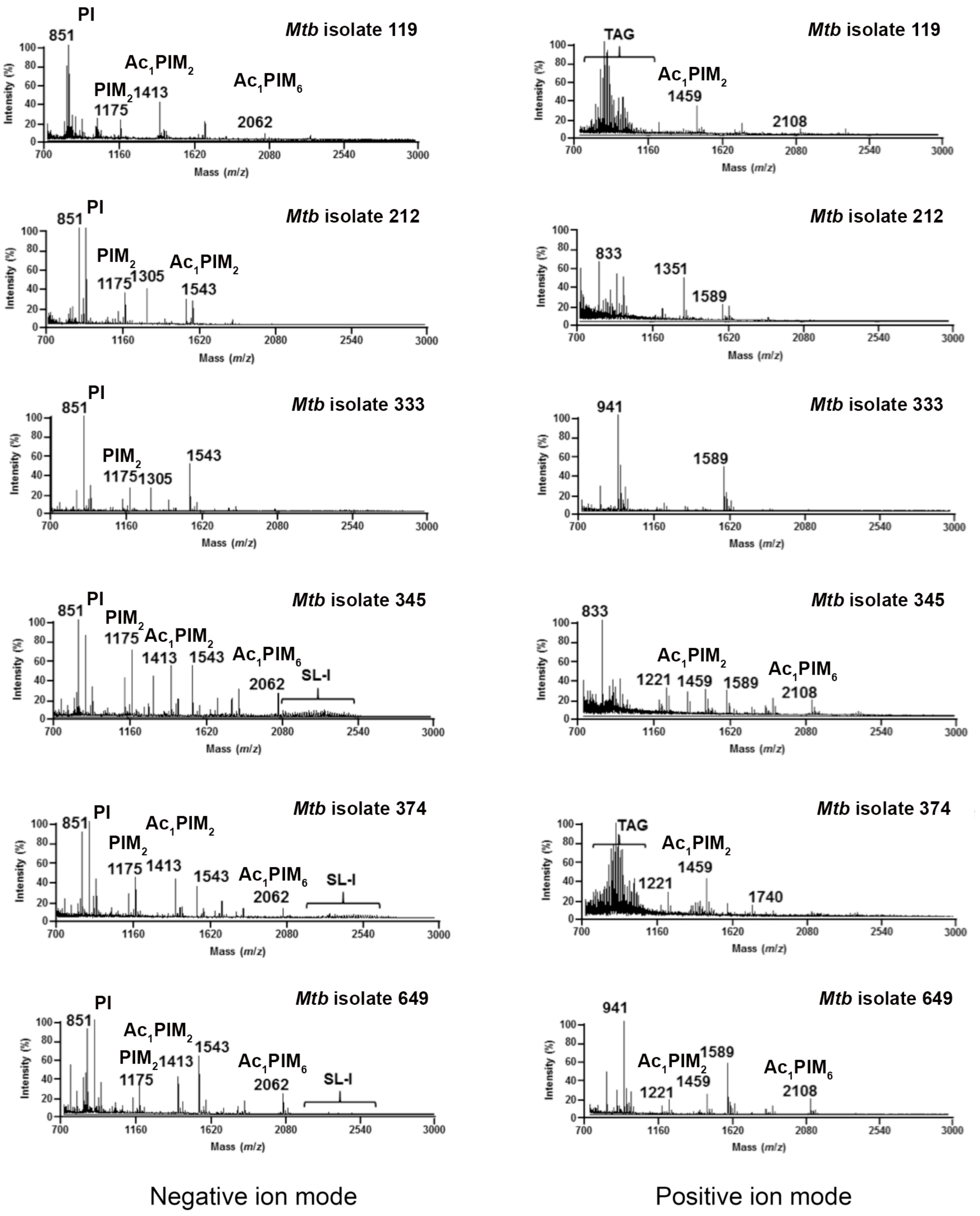

g

Mtb Lineage 3.

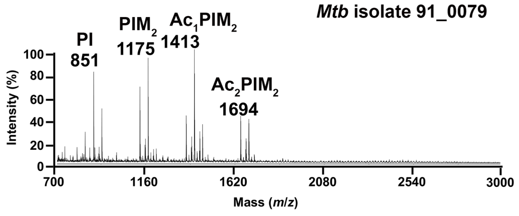

Negative ion mode

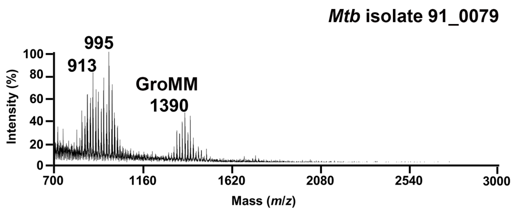

Positive ion mode

h

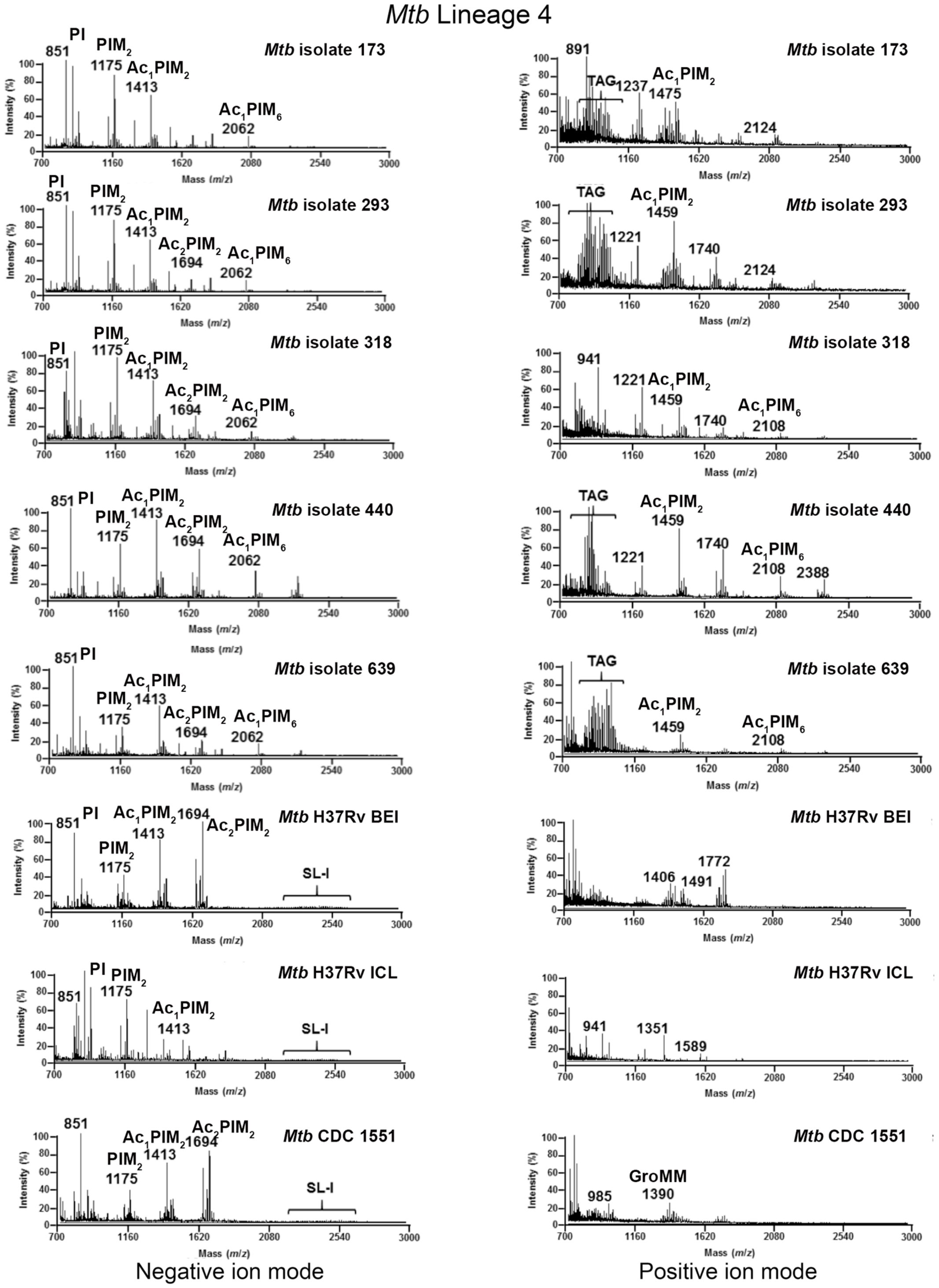
Mtb Lineage 4
Mtb isolate 173
PI
851
PIM2
1175
Ac1PIM2
1413
Ac1PIM6
2062
891
TAG
1237
Ac1PIM2
1475
2124
Mtb isolate 293
851
PIM2
1175
Ac1PIM2
1413
Ac2PIM2
1694
Ac1PIM6
2062
TAG
Ac1PIM2
1459
1221
1740
2124
Mtb isolate 318
PI
851
PIM2
1175
Ac1PIM2
1413
Ac2PIM2
1694
Ac1PIM6
2062
941
1221
Ac1PIM2
1459
1740
Ac1PIM6
2108
Mtb isolate 440
851 PI
PIM2
1175
Ac1PIM2
1413
Ac2PIM2
1694
Ac1PIM6
2062
TAG
1221
Ac1PIM2
1459
1740
Ac1PIM6
2108
2388
Mtb isolate 639
851 PI
PIM2
1175
Ac1PIM2
1413
Ac2PIM2
1694
Ac1PIM6
2062
TAG
Ac1PIM2
1459
Ac1PIM6
2108
Mtb H37Rv BEI
851 PI
PIM2
1175
Ac1PIM2
1413
1694
Ac2PIM2
SL-I
1406
1491
1772
Mtb H37Rv ICL
851
PI
PIM2
1175
Ac1PIM2
1413
SL-I
941
1351
1589
Mtb CDC 1551
851
PIM2
1175
Ac1PIM2
1413
Ac2PIM2
1694
SL-I
GroMM
1390
985
Intensity (%)
Mass (m/z)
Negative ion mode
Positive ion mode

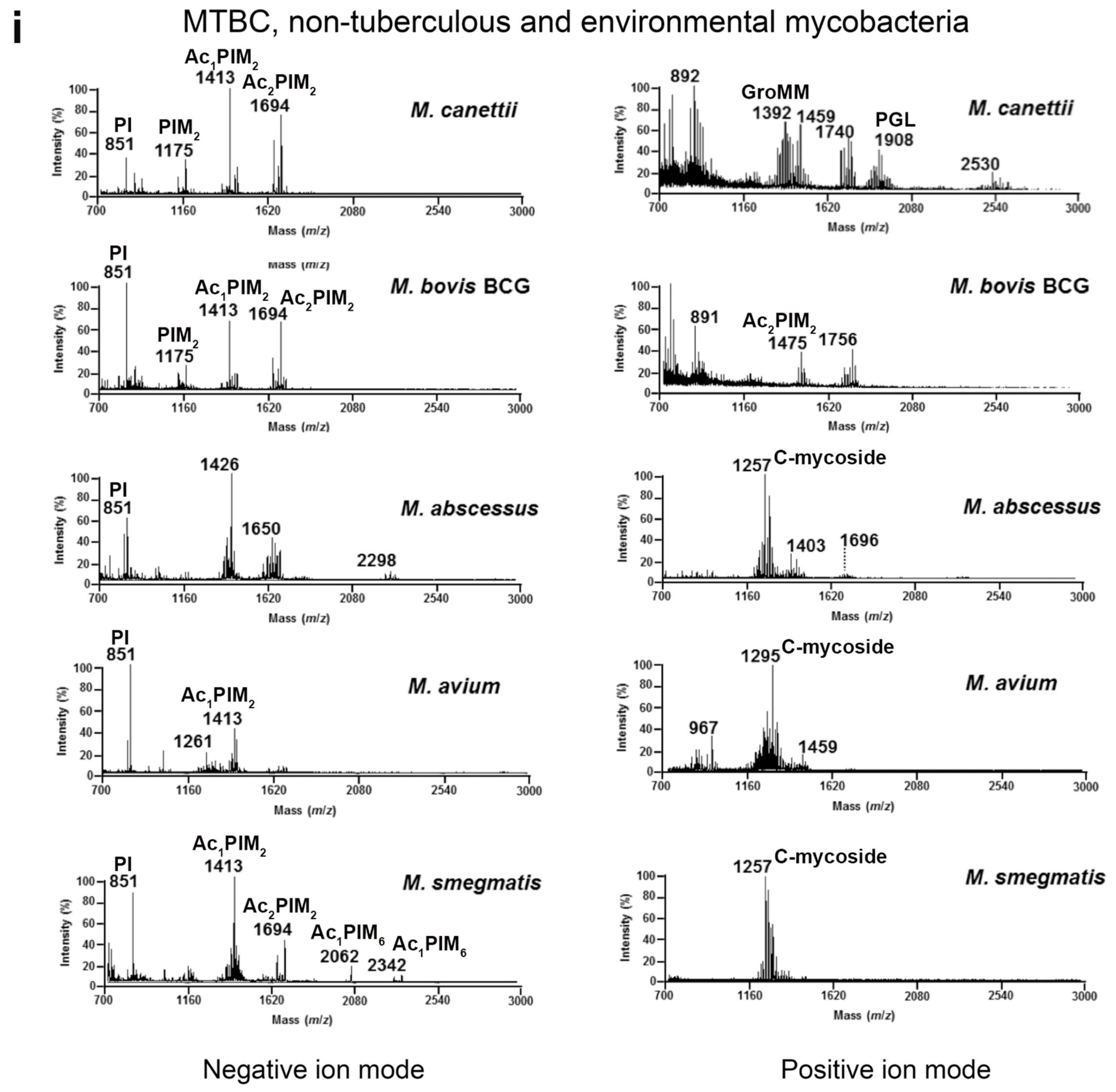

Supplementary Figure 4: Thin-layer chromatograms and MS analysis of lipids from different mycobacteria. TLCs of lipids from *Mtb* clinical isolates. TLCs were run in four different solvent systems. Petroleum Ether/Diethyl Ether 95:5 (v/v) (a); $CHCl_3$/MeOH 9:1 (v/v) (b); $CHCl_3$/MeOH 8:2 (v/v) (c) and $CHCl_3$/MeOH/H_2O 60:25:4 (v/v/v) (d). The migration of specific lipid species is indicated. TDM, trehalose dimycolate; TMM, trehalose monomycolate; CL, cardiolipin; PE, phosphatidyl-ethanolamine; Ac_4PIM_2, tetra-acyl-phosphatidyl-*myo*-inositol-dimannosides; Ac_3PIM_2, tri-acyl-phosphatidylinositol dimannosidase; DIM A, phthiocerol dimycocerosates, DIM B, phthiodiolone dimycocerosates; TAG, triacylglycerol; GroMM, glycerol monomycolate. Panels e-i. MALDI lipid fingerprinting of mycobacteria. MALDI-ToF mass spectrometry of mycobacterial lipids in negative ion mode (left hand panels) and positive ion mode (right hand panels). e, *Mtb* lineage 1; f, *Mtb* lineage 2; g, *Mtb* lineage 3; h, *Mtb* lineage 4; i, MTBC, non-tuberculous and environmental mycobacteria. TAG, Triacylglycerol; SL-1, sulfolipid-1; PI, phosphatidylinositol; PIM_2, di-acyl-phosphatidyl-*myo*-inositol-dimannosides; Ac_1PIM_2, tri-acyl-phosphatidyl-*myo*-inositol-dimannosides; Ac_2PIM_2, tretra-acyl-phosphatidyl-*myo*-inositol-dimannosides; Ac_1PIM_6, tri-acyl-phosphatidyl-*myo*-inositol-hexamannosides; Ac_2PIM_2, tretra-acyl-phosphatidyl-*myo*-inositol-hexamannosides; GroMM, glycerol monolycolate; PGL, phenol glycolipid. Molecular masses are indicated. Data are representative of two independent experiments. Source data are provided as a Source data file.

Reference

1. Pandey, S.D., *et al.* Iron-regulated protein HupB of Mycobacterium tuberculosis positively regulates siderophore biosynthesis and is essential for growth in macrophages. *J Bacteriol* **196**, 1853-1865 (2014).
