# Supplementary figures and images for: Inhibition of the Niemann-Pick C1 protein is a conserved feature of multiple strains of pathogenic mycobacteria
Source: Nat Commun. 2022 Sep 9;13:5320. doi: 10.1038/s41467-022-32553-0 (PMC9463166; doi:10.1038/s41467-022-32553-0)

**Fig 3f Blot**

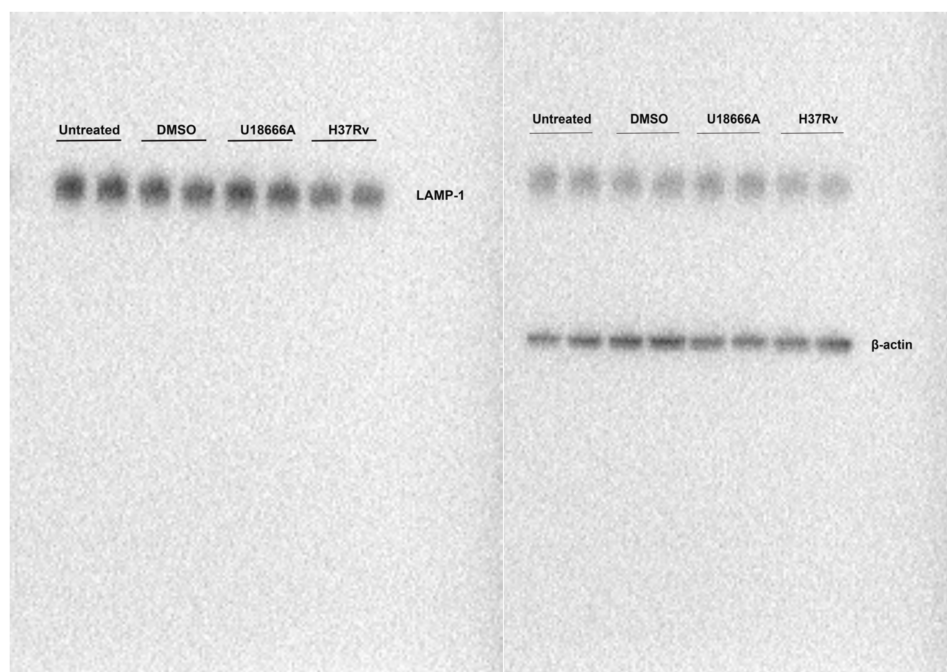

**Full size blots from Fig 3f.**

Supplement: Supplementary file 4 — Source Data [file 41467_2022_32553_MOESM4_ESM.zip › Fig 3f full blot.pdf]

Fig 4A blot.

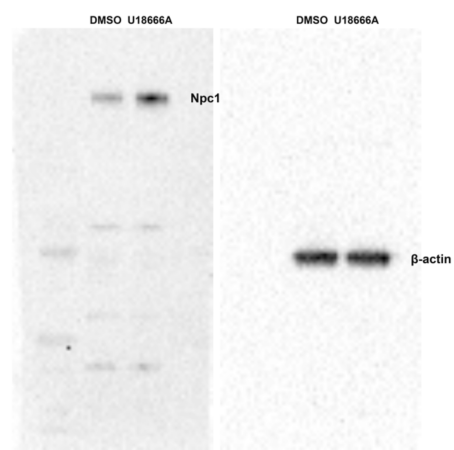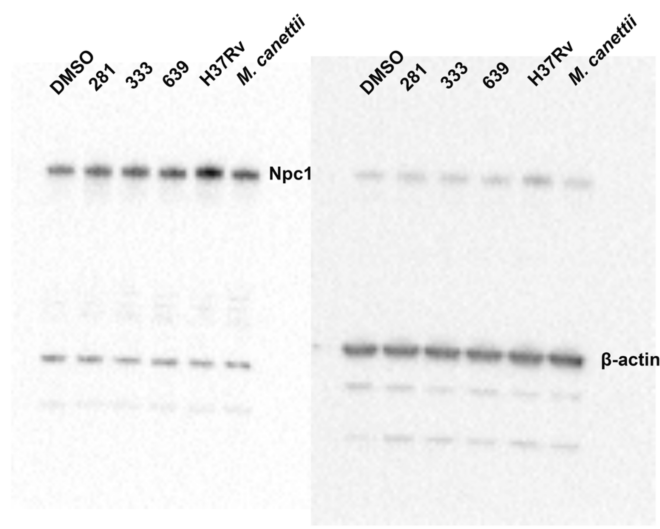

Full size blots from Fig 4A.

Supplement: Supplementary file 4 — Source Data [file 41467_2022_32553_MOESM4_ESM.zip › Fig 4a full blot.pdf]
